# Detailed causality between coronavirus disease‐2019 and atrial fibrillation

**DOI:** 10.1002/clc.24258

**Published:** 2024-03-25

**Authors:** Naoya Kataoka, Teruhiko Imamura

**Affiliations:** ^1^ Second Department of Internal Medicine University of Toyama Toyama Japan

To the Editor,

Niu et al. investigated the clinical implication of atrial fibrillation (AF) in patients with coronavirus disease‐2019 (COVID‐19).^1^ According to their findings, AF was prevalent in patients hospitalized for COVID‐19 and was associated with worse in‐hospital mortality, more disease‐related complication, and increased healthcare utilization. Several concerns have been raised.

COVID‐19 is a systemic inflammatory disease that induces various cardiovascular conditions.^2^ AF may be triggered by this inflammatory cascade. Consequently, the presence of AF may be a confounding factor for in‐hospital mortality rather than a prognostic factor. Did the authors observe obvious differences in the severity and inflammatory status of COVID‐19 between those with and without AF?

It is widely acknowledged that AF is a robust predictor of cardiovascular diseases and mortality.^3^ Did the authors identify any distinct profiles in individuals who had both COVID‐19 and AF when compared to those with AF alone?

In the authors' study, the incidence of ventricular arrhythmia was higher in individuals with AF.^1^ However, the detailed types of ventricular arrhythmia remain uncertain. For instance, did the authors include premature ventricular contractions? It is plausible that the dominant causes of in‐hospital cardiac arrest were ventricular tachycardia and ventricular fibrillation. Ventricular fibrillation is often encountered in younger patients with acute coronary syndrome, while extra‐cardiac abnormalities such as anemia and advanced age are associated with pulseless electrical activity.^4^ Could the authors provide more clarity on the relationship between COVID‐19, AF, and cardiac arrest?
